# Emerging biotechnologies and non-thermal technologies for winemaking in a context of global warming

**DOI:** 10.3389/fmicb.2023.1273940

**Published:** 2023-10-06

**Authors:** Piergiorgio Comuzzo, Juan Manuel del Fresno, Sabrina Voce, Iris Loira, Antonio Morata

**Affiliations:** ^1^Dipartimento di Scienze Agroalimentari, Ambientali e Animali, Università degli Studi di Udine, Udine, Italy; ^2^enotecUPM, Universidad Politécnica de Madrid, Madrid, Spain

**Keywords:** non-*Saccharomyces* yeasts, *Lachancea thermotolerans*, *Hanseniaspora* spp., *Metschnikowia pulcherrima*, pH control, alcohol reduction, bioprotection, SO_2_ alternatives

## Abstract

In the current situation, wine areas are affected by several problems in a context of global warming: asymmetric maturities, pH increasing, high alcohol degree and flat wines with low freshness and poor aroma profile. The use of emerging biotechnologies allows to control or manage such problems. Emerging non-*Saccharomyces* as *Lachancea thermotolerans* are very useful for controlling pH by the formation of stable lactic acid from sugars with a slight concomitant alcohol reduction. Lower pH improves freshness increasing simultaneously microbiological stability. The use of *Hanseniaspora* spp. (specially *H. vineae* and *H. opuntiae*) or *Metschnikowia pulcherrima* promotes a better aroma complexity and improves wine sensory profile by the expression of a more complex metabolic pattern and the release of extracellular enzymes. Some of them are also compatible or synergic with the acidification by *L. thermotolerans*, and *M. pulcherrima* is an interesting biotool for reductive winemaking and bioprotection. The use of bioprotection is a powerful tool in this context, allowing oxidation control by oxygen depletion, the inhibition of some wild microorganisms, improving the implantation of some starters and limiting SO_2_. This can be complemented with the use of reductive yeast derivatives with high contents of reducing peptides and relevant compounds such as glutathione that also are interesting to reduce SO_2_. Finally, the use of emerging non-thermal technologies as Ultra High-Pressure Homogenization (UHPH) and Pulsed Light (PL) increases wine stability by microbial control and inactivation of oxidative enzymes, improving the implantation of emerging non-*Saccharomyces* and lowering SO_2_ additions.

**GRAPHICAL ABSTRACT:**
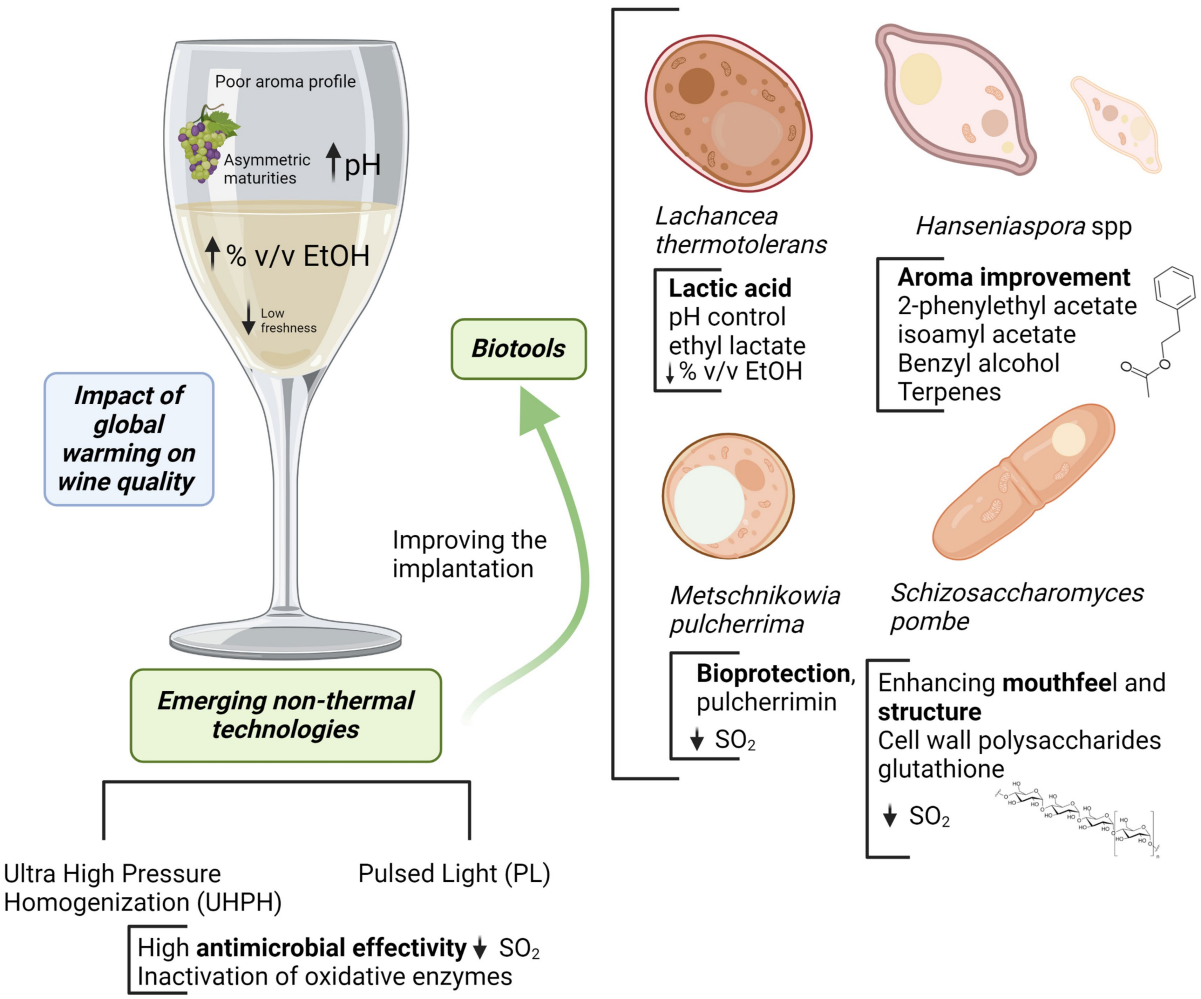

## Highlights

- Acidifying non-*Saccharomyces* yeasts: *Lachancea thermotolerans.*
- Aroma improvement with apiculate yeasts from the genus *Hanseniaspora* spp.
- Bioprotection, aroma enhancement and biocompatibility by *Metschnikowia pulcherrima.*
- Yeast polysaccharides and reductive compounds from cell walls and aging on lees. The use of *Schizosaccharomyces pombe* and *Schizosaccharomyces japonicus.*
- Control of malolactic fermentation with fumaric acid.
- Emerging non-thermal technologies to improve the implantation of non-*Saccharomyces* yeasts and to control oxidative enzymes.

## Introduction

Global warming is already clearly affecting grape ripening by modifying the composition and affecting polyphenol contents, freshness, acidity, pH, sugars, stability, sensory balance, aroma and color (Jones et al., 2005; Mozell and Thach, 2014; Drappier et al., 2019; Gutiérrez-Gamboa et al., 2021). Additionally, global warming is increasing the water stress in vines due to lower rainfall in many regions especially during the fruit growth and increasing pests and diseases (Jones and Alves, 2012; Sgubin et al., 2022).

The global warming in some regions has produced an improvement in quality but it looks that is reaching its peak (Gambetta and Kurtural, 2021). A global temperature increase of 0.3–1.7°C is expected in the coming years (Drappier et al., 2019), therefore, the undesirable effects of temperature on grape maturity will be worst in the near future. Several viticultural techniques have been proposed as a tool to improve grape maturity in a context of global warming. Some of them can produce a 15-day delay in maturity by better adapting vine physiology and growth cycle to climatic conditions (Gutiérrez-Gamboa et al., 2021). Among them, the following can be considered as quite effective: severe shoot trimming, minimal pruning, leaf removal, late pruning and specially forced regrowth that can delay maturity by up to 2 months (Gutiérrez-Gamboa et al., 2021).

Different physicochemical options can be used to control sugars and acidity levels, such as the use of cation exchange resins (Lasanta et al., 2012) or electrodialysis with bipolar membranes to increase acidity and therefore reduce pH, or membrane technologies (i.e., reverse osmosis or nanofiltration) to reduce sugar content (El Rayess and Mietton-Peuchot, 2016).

But there are also many possibilities to control some of the most important effects of global warming in the winery by using fermentation biotechnologies. Concerning the loss of acidity due to high temperatures and the consequent effect on pH, freshness, and wine stability, a powerful tool is the use of acidifying yeasts that can reduce pH during fermentation by producing organic acids from sugars. Among them, the malic acid producers *Saccharomyces cerevisiae* (Sc) strains (Yéramian et al., 2007; Vion et al., 2023) and especially the lactic acid producers *Lachancea thermotolerans* (Lt) have been studied (Comitini et al., 2011; Morata et al., 2018; Porter et al., 2019a). The production of malic acid after a breeding process by selected *S. cerevisiae* yeasts can be higher than 3 g/L (Vion et al., 2023), with an impact on the sensory perception of the wines. The main issue concerning the production of malic acid is linked to its instability; in addition, it can be metabolized by lactic acid bacteria producing colloidal haze in the bottle. Regarding Lt, it has been reported that one specific strain produced more than 16 g/L of lactic acid (Banilas et al., 2016), but it is easy to find strains able to produce 1–5 g/L under enological conditions (Morata et al., 2022a). The use of Lt also determines a slight reduction in alcohol content (Ciani et al., 2016; Morata et al., 2019a; Hranilovic et al., 2021), being the latter a concomitant problem in warm areas.

## Acidifying non-*Saccharomyces* yeasts: *Lachancea thermotolerans*

*Lachancea thermotolerans* (Lt), formerly known as *Kluyveromyces thermotolerans* or *Zygosaccharomyces thermotolerans*, is a *Saccharomycetaceae* yeast that was first described in 1932 (Kurtzman, 2003). It is named *Lachancea* in honor to Dr. Marc-André Lachance, University of Western Ontario, Canada. The morphology is similar to that of *Saccharomyces cerevisiae* with an ovoid or spheroidal shape and multilateral budding during asexual reproduction and forms 1–4 ascospores by sexual reproduction (Kurtzman, 2003). Lt has a medium fermentative power reaching 5–9% v/v of ethanol (Morata et al., 2018), so it should be used in coinoculation or sequential fermentation with Sc to complete alcoholic fermentation and produce dry wines. Lt has also been described as a low volatile acidity producer (0.1–0.5 g/L) (Kapsopoulou et al., 2007; Comitini et al., 2011; Aponte and Blaiotta, 2016; Morata et al., 2019a) and moreover it is able to reduce volatile acidity under aerobic conditions (Vilela-Moura et al., 2008), with a moderate formation of ethyl acetate similar to Sc (Morata et al., 2019a). Moreover, even if Lt is sensitive to sulfites (Comitini et al., 2011), some strains can produce effective acidification at 25–75 mg/L of total SO_2_ (Vaquero et al., 2020), and its growth is affected by low fermentation temperatures (Vaquero et al., 2020).

Its main application in emerging wine biotechnology is acidification and pH control by metabolizing sugars to lactic acid with the use of lactate dehydrogenase enzymes (LDH). Pyruvate can be reduced to lactate by LDH as an alternative pathway to recover NAD+, thus reducing ethanol formation (Hranilovic et al., 2018). Bioproduction of lactic acid in Lt is related to the expression of three lactate dehydrogenase enzymes (LDH) and appears to be unaffected by the expression of alcohol dehydrogenases (ADH) (Sgouros et al., 2020), because of the similar expression of ADH in high and low lactic acid producing strains. LDH2 is up-regulated in high producing strains (Sgouros et al., 2020). Moreover, it has been observed in several strains that LDH2 and LDH3 are organized in tandem and LDH1 is located elsewhere (Gatto et al., 2020). There are also two lactate permeases involved in lactate excretion, JEN1 in a single copy and 2 sequences of ADY2 (Gatto et al., 2020).

The effect of Lt is very powerful, and it is easy to decrease the pH of 0.1–0.5 units, depending on the fermentation time; however, most of the acidification is done at the beginning of fermentation, before day 6 (Morata et al., 2018). Many Lt strains can degrade malate but some of them are also able to produce small amounts of malic acid, up to 0.3 g/L (Hranilovic et al., 2018).

Lt has shown a good ability to control pH in real musts or crushed grapes from several *Vitis vinifera* L. white (Albariño, Airen, Vilana, Treixadura, Viognier) and red varieties (Tempranillo, Mencía, Merlot, Cabernet sauvignon) (Balikci et al., 2016; Morata et al., 2019a; Binati et al., 2020; Blanco et al., 2020; Sgouros et al., 2020; Vaquero et al., 2020, 2021a; Hranilovic et al., 2021, 2022; Zhang et al., 2023). The pH reduction is variable depending on several factors, but especially on the strain, and usually the reduction can range between 0.3 and 0.5 pH units (Table 1). The concomitant alcohol reduction has ranged from 0.2–0.5% v/v (Table 1). The scale up has been from 0.2–3 L in the laboratory to 60 L-400hL at pilot plant scale (Table 1).

**Table 1:** Relevant literature results concerning wine acidification by fermentation with *Lachancea thermotolerans* in sequential fermentation or coinoculation with *S. cerevisiae* (Sc).

*Vitis vinifera* L. grape variety	Scale	Total SO_2_ (mg/L)	Lactic acid (g/L)	pH reduction	Volatile acidity (g/L)	Alcohol reduction (% v/v)	Fermentation biotechnology	strains	Reference
White									
Emir	0.8 L must	-	0.4–1.28	0.0	0.53–0.73	-	Sequential and coinoculation with Sc	CBS2860	Balikci et al. (2016)
Albariño	30 L must	30	2.7	0.3	0.4	0.2	Sequential with Sc	L31	Morata et al. (2019a)
Airen	1 L must	25–75	0.2–4	0.1–0.5	-	-	Sequential with Sc	Laktia, Concerto, L31, A54, F108, F111	Vaquero et al. (2020)
Vilana	2.2 L must	30	<0.6–5.5	0.1–0.2	0.52	0–0.3	Sequential and coinoculation with Sc	P-HO1	Sgouros et al. (2020)
Treixadura	1 L	50	0.2	0.05	0.36–0.39	-	Sequential with Sc	Lt93	Blanco et al. (2020)
Airen	30 L must	100	0.9–1	0.2–0.3	0.17–0.25	0.1–0.5	Sequential with Sc	L31, Laktia	Vaquero et al. (2021)
Viognier	3 L must	60	0.1–5.2	0.1–0.5	0.3–0.4	0–0.3	Sequential and coinoculation with Sc	Levulia, Concerto, Laktia, ISVV Ltyq 25, UNIFG 18	Hranilovic et al. (2021)
Rose									
Pinot grigio	0.2 L	-	0.5–4.4	-	0.19–0.26	0.1–0.35	Sequential	COLC27, DESP53, SOL13	Binati et al. (2020)
Red									
Tempranillo	800 Kg crushed grape	30	6.6	0.5	0.4	0.2	Sequential with Sc	L31	Morata et al. (2019a)
Mencía	1 L	50	7.1–7.2	0.2	0.36–0.42	0.5–0.7	Sequential with Sc	Lt93	Blanco et al. (2020)
Merlot	3Kg crushed grape	50	0.6–8.1	0.0–0.5	0.21–0.67	0.9	Sequential and coinoculation with Sc	Levulia, Concerto, Laktia, ISVV Ltyq 25, UNIFG 18	Hranilovic et al. (2021)
Cabernet sauvignon	60 L grape must	60	5.0–7.0	0.5	0.4–0.6	1.9	Sequential and coinoculation with Sc	CVE-LT1 CGMCC NO.15161	Zhang et al. (2023)
Cabernet sauvignon	400hL grape must	60	2.2–2.8	-	0.7	-	Sequential and coinoculation with Sc	CVE-LT1 CGMCC NO.15161	Zhang et al. (2023)

The high formation of lactic acid during fermentation with Lt can also have another positive effect in warm areas that is the by-product inhibitory effect on malolactic fermentation (MLF) (Morata et al., 2020a; Snyder et al., 2021), resulting in the preservation of malic acid that keeps the wines fresher and less flat. A strong inhibitory effect on MLF has been observed when lactic acid levels are 4 g/L or higher (Morata et al., 2020a), and it is even delayed at concentrations above 2 g/L. Moreover, it should be considered that lactic acid concentration is stable and cannot be degraded by microorganisms, so it preserves the wines from undesirable MLF during wine storage, aging on lees or bottle aging in sparkling wines.

**Table 2:** Main features of UHPH and PL in wines.

Technique	Ultra High-Pressure Homogenization (UHPH)	Reference	Pulsed Light (PL)	Reference
Features				
Control of vegetative cells	Highly effective > 6 log reductions	Loira et al. (2018a)	Highly effective	Santamera et al. (2020) and Vargas-Ramella et al. (2021)
Elimination of spores	Yes, depending on in-valve temperature	Zamora and Guamis (2015) and Morata and Guamis (2020)	Yes, the color of the spore can influence the sensitivity	Gómez-López et al. (2007)
Inactivation mechanisms	Impact and shear efforts	Zamora and Guamis (2015) and Morata and Guamis (2020)	UV 254 nm, photochemical effect. Thermolysis	Gómez-López et al. (2007) Santamera et al. (2020) and Vargas-Ramella et al. (2021)
Continuous processing	Yes. For liquids: grape must and wine	Morata and Guamis (2020)	Yes. For solids: grapes. Surface irradiation. For liquids: treatment depth must be <1 mm	Gómez-López et al. (2007) and Santamera et al. (2020)
Temperature increase	70–80°C in valve 0.2 s. Quickly reduced after expansion.	Morata and Guamis (2020) and Bañuelos et al. (2020)	3–4°C	Santamera et al. (2020)
Inactivation of oxidative enzymes (PPOs)	Yes, by enzyme denaturalization or unfoldment	Bañuelos et al. (2020)	Yes, by photothermal effect	Gómez-López et al. (2007) and Bhagat and Chakraborty (2022)
Antioxidant activity	Preserved	Loira et al. (2018a) and Bañuelos et al. (2020)	Preserved, small reduction 6–15%	Chakraborty et al. (2020) and Bhagat and Chakraborty (2022)
Control of browning	Positive	Bañuelos et al. (2020)	Slightly by overheating and oxidation	Gómez-López et al. (2007)
Effect on anthocyanins	Not affected	Vaquero et al. (2022)	Scarcely affected some photodegradative oxidation	Escott et al. (2017) , Chakraborty et al. (2020), and Bhagat and Chakraborty (2022)
Effect on terpenes	Not affected	Bañuelos et al. (2020)	Decrease the content in wines	Pérez-López et al. (2020)

The sensory impact of biological acidification by Lt is positive, producing a ‘citric freshness’ in the wines (Morata et al., 2020b), without ‘dairy notes’ that are more typical in MLF due to the formation of carbonyl metabolites such as diacetyl or acetoin. Lt produces these compounds at low concentrations like *Saccharomyces cerevisiae*.

In terms of aroma contribution, Lt has been described as a moderate producer of higher alcohols, with influence on aroma modulation by the production of floral acetate esters such as 2-phenylethyl acetate (Comitini et al., 2011; Gobbi et al., 2013; Morata et al., 2019a). Lt also produces high amounts of ethyl lactate that can be 30-folds higher than in Sc fermentations (Hranilovic et al., 2021) due to the formation of high levels of lactic acid. Furthermore, some strains are able to release terpenes and thiols as 4MMP and 3MH (Zott et al., 2011) by potential *β*-glucosidase and carbon-sulfur lyase activities (Rosi et al., 1994; Zott et al., 2011; Porter et al., 2019b). Therefore, even when the main effect of the use of Lt in wines from areas affected by global warming is on the control of pH and alcoholic degree, a positive modulation of aroma can also be obtained by the formation of positive floral and fruity esters or the release of bonded thiols and terpenes. Moreover, the acidification produced by Lt increases the amount of molecular SO_2_ by pH effect, helping to reduce the overall doses and increasing the effectiveness of its antimicrobial and antioxidant functions (Morata et al., 2021). The selection and industrial scale-up of this species, including production as dry yeast, is well designed and optimized, making Lt a good candidate for industrial production (Morata et al., 2022b).

## Aroma improvement with apiculate yeasts from the genus *Hanseniaspora* spp.

Apiculate yeasts of the genus *Hanseniaspora* usually predominate on grape skins at maturity and, in the early stages of spontaneous fermentation, they dominate up to an alcohol level of 4–6% v/v (Moreira et al., 2011).

*Hanseniaspora* spp. have apiculate shape, like lemon, with polar budding (Martin et al., 2018). These yeasts have been traditionally considered as undesired and excluded in the fermentation using SO_2_, because of their potential effect on volatile acidity and ethyl acetate formation. However, several species, depending on the strain, produce moderate volatile acidity, some of them (e.g., *Hanseniaspora vineae* – Hv) even lower than Sc (del Fresno et al., 2021a). Additionally, *Hanseniaspora* spp. have been frequently described as overproducers of floral and fruity acetate esters such as 2-phenylethyl acetate or isoamyl acetate, positively improving flat wines from neutral varieties (Moreira et al., 2011). Moreover, the high glucosidase activity observed in some strains (Testa et al., 2020) may contribute to enhance the varietal characteristics of wines (Lombardi et al., 2018). Several recent reviews have highlighted the usefulness and positive impact of these species in wine technology, including aroma improvement and the effect on body and structure (Martin et al., 2018; van Wyk et al., 2023). Therefore, *Hanseniaspora* can be very useful for improving flat wines from regions affected by global warming, also having additional interesting applications in biocontrol (van Wyk et al., 2023).

Hv is a very interesting species from an enological point of view and described as a ‘friendly’ yeast (Carrau and Henschke, 2021), because of its ability to intensify the floral and fruity notes in wines by a high acetylation capacity and a highly developed phenylpropanoids pathway (Valera et al., 2021; Carrau et al., 2023), compared to other species such as *H. uvarum*. It is also convenient to use due to its good fermentative power, easily reaching 8–10% v/v ethanol, and with a production of volatile acidity lower than many Sc strains, often with values below 0.4 g/L (Martin et al., 2018; del Fresno et al., 2021a). Hv produces higher levels of 2-phenylethyl acetate and benzyl alcohol (Viana et al., 2011; Valera et al., 2021). Benzyl alcohol can be synthetized *de novo* by Hv (Martin et al., 2016) and the average content in several vinifications is 14-folds that obtained by Sc (Carrau et al., 2023). Hv yields high terpene contents in some must fermentations (x3 on average compared to Sc) (del Fresno et al., 2021a). The production of some specific spice compounds such as safranal in Hv above its sensory threshold has also been described (del Fresno et al., 2022). Protective effects on color has also been observed with improved hue parameters that can be representative of a lower oxidation in rose wines (del Fresno et al., 2021b).

*Hanseniaspora opuntiae* (Ho) is also an apiculate yeast but smaller than Hv (Vaquero et al., 2022). It can promote the release of some terpenes as citronellol (del Fresno et al., 2022; Badura et al., *2023*) and has been described as a good producer of floral and fruity acetate esters (Bourbon-Melo et al., 2021; del Fresno et al., 2022), conferring floral notes in wines (Luan et al., 2018), with moderate volatile acidity and low levels of ethyl acetate (del Fresno et al., 2022). However, the fermentative power is lower than in Hv usually reaching 4–6% v/v ethanol depending on strains and fermentation conditions. A good compatibility with Lt has been observed to achieve good acidification as well as suitable release of aromatic esters (Vaquero et al., 2022). When Hv is used together with Lt, the high fermentative performance of Hv strongly decreases the acidification capacity of Lt (Vaquero et al., 2021). As observed for Hv and other *Hanseniaspora* spp., Ho also produces wines with good body, volume and a softer mouthfeel (Vaquero et al., 2022).

## Bioprotection, aroma enhancement, and biocompatibility by *Metschnikowia pulcherrima*

*Metschnikowia pulcherrima* (Mp) is a globous or ellipsoidal multipolar budding yeast that evolves to spherical in adult cells due to the accumulation of large amounts of fatty compounds in the vacuole (Morata et al., 2019b). Several reviews have focused on the properties, characteristics and winemaking applications of Mp (Morata et al., 2019b; Sipiczki, 2020). It typically increases the fruity profile in wines and produces a positive sensory impact (Varela et al., 2017, 2021; Binati et al., 2020). In addition, many strains express *β*-glucosidase and *β*-lyase activities with remarkable intensity, thus promoting the release of free terpenes and volatile thiols in aromatic varieties (Barbosa et al., 2018). Some strains can be used to reduce the alcohol content of wines (Hranilovic et al., 2020). This species produces moderate or low levels of volatile acidity and H_2_S (Barbosa et al., 2018). Ethanol tolerance is quite good reaching 3–4%v/v in single fermentation (Barbosa et al., 2018), and viable cells of Mp can be found in the middle-end of alcoholic fermentation.

Mp has been considered an interesting yeast species for bioprotection with an effective antimicrobial effect against some non-*Saccharomyces* yeasts, but with good compatibility with Sc (Oro et al., 2014; Di Gianvito et al., 2022; Canonico et al., 2023). The antimicrobial and antioxidant activity of this species is mainly based on the production of pulcherrimin (Morata et al., 2019b; Sipiczki, 2020) and the effect on iron chelation. Prefermentative use of Mp has been suggested as an alternative to control microorganisms and to avoid or reduce SO_2_ (Simonin et al., 2020; Windholtz et al., 2021a,b; Agarbati et al., 2023). A non-negligible production of glutathione (GSH) was also observed for *Metschnikowia* spp. during the growth phase (Lemos Junior et al., 2021); during sequential fermentation, some strains may increase the final GSH content in wine up to 10 mg/L (Binati et al., 2021), potentially reducing oxidation risks and reducing SO_2_ requirements.

Mp has shown also very good biocompatibility and synergistic behavior concerning the acidification when used with Lt (Vaquero et al., 2021; Escott et al., 2022), together with a positive sensory impact. Therefore, the simultaneous use of Lt/Mp starters increases the acidification and the low pH promotes a higher proportion of molecular SO_2_, while producing a natural biocontrol on microbial populations, and improving the aroma profile.

## Yeast polysaccharides and reductive compounds from cell walls and aging on lees: the use of *Schizosaccharomyces pombe*

Yeast derivatives, by-products and yeast lees during aging on lees are being widely used as additives to improve wine quality, by increasing mouthfeel and structure and by softening tannin astringency, but also to stabilize and clarify wines (Morata et al., 2019c; Vejarano, 2020; Rigou et al., 2021). The effect of lees and yeast by-products on the aromatic fraction has also been studied (Comuzzo et al., 2006, 2011; Loira et al., 2013), as well as the absorption of aroma and off-flavors by lees (Chassagne et al., 2005), or the application of lees as drivers of wood aroma has also been observed (Palomero et al., 2015).

To speed and enhance the effect of aging on lees or yeast derivatives, the use of non-*Saccharomyces* yeasts has been an innovative and powerful tool (Morata et al., 2019c; Vejarano, 2020). Some non-*Saccharomyces* yeasts such as *Schizosaccharomyces pombe* (Sp) or *Schizosaccharomyces japonicus* (Sj) have a special aptitude to release higher cell wall polysaccharide contents in a shorter time and with a positive impact on wine quality (Palomero et al., 2009; Domizio et al., 2017, 2018; Loira et al., 2018b; Portaro et al., 2022). The polysaccharide release capacity of several Sj strains is even higher than that of Sp (Domizio et al., 2017); even if their profiles show similarities, some differences in terms of galactose/mannose ratio have been observed. Both yeast species show interesting properties to be used in aging on lees and in the preparation of yeast derivatives. Polysaccharides of Sj have also produced a positive effect on the control of protein haze in wines (Millarini et al., 2020). The use of these biotechnological products derived from Sc or new species such as Sp or Sj is an interesting tool to improve wine volume and tannin integration in unbalanced grapes from warm areas.

The use of yeast derivatives rich in nitrogen reducing compounds and glutathione (GSH) is another key application of these additives that is especially relevant for reducing the use of SO_2_ as antioxidant (Rodríguez-Bencomo et al., 2014; Comuzzo et al., 2015; Bahut et al., 2019; Pons-Mercadé et al., 2021; Nioi et al., 2022). Currently, all the yeast derivatives marketed for winemaking use are from *Saccharomyces* spp. However, besides Sj and Sp (discussed above), other non-*Saccharomyces* strains might be exploited for this purpose. Different *Hanseniaspora* yeasts for instance, showed a relevant production of polysaccharides, thiol molecules and GSH during growth and after autolysis, in some cases even higher than certain *Saccharomyces* strains (Voce et al., 2022). The possibility to use yeast derivatives from non-*Saccharomyces* yeasts in winemaking is currently under discussion (step 3 out of 7) at the International Organization of Vine and Wine (OIV).^1^

## Control of malolactic fermentation with fumaric acid

Recently, fumaric acid (FA) has been approved by the OIV to control MLF at a maximum dose of 600 mg/L (OIV, 2021), thanks to the inhibition of lactic acid bacteria (LAB), consequently preserving the malic acidity of wines (Cofran and Meyer, 1970; Tchelistcheff et al., 1971; Pilone et al., 1974). The control of LAB protects wines and helps to reduce SO_2_ levels. Additionally, FA at the allowed dose of 0.6 g/L can lower the pH of 0.05–0.1 units, also depending on the buffering power of the wine (Morata et al., 2023). FA is a stronger acidifier than tartaric acid (Gancel et al., 2022) and currently this additive is under evaluation by OIV also for wine acidification at 2–3 g/L. At the allowed dose, FA has a stronger inhibitory effect, even controlling and stopping an ongoing MLF with 60% malic acid degradation (Morata et al., 2020a). The inhibitory effect against other bacteria has also been published (Barnes and Karatzas, 2020), with an intense effect against acetic acid bacteria. Therefore, FA is a powerful tool to preserve and increase acidity in wines from warm areas. Its use can be complementary to malic acid-producing Sc yeasts or to Lt.

## Emerging non-thermal technologies to improve the implantation of non-*Saccharomyces* yeasts and to control oxidative enzymes

The main drawback of most non-*Saccharomyces* yeasts is the lower fermentative power and the weaker competitiveness compared to Sc, which makes it necessary to use them in sequential or mixed fermentations, and to facilitate their implantation by must processing. Non-thermal technologies are very interesting to facilitate the implantation of non-*Saccharomyces* yeasts because of their high antimicrobial effectiveness and the mild effect on the sensory quality of grape and must (Morata et al., 2017). Among them, two techniques are particularly interesting for their efficacy and protective effect on sensory quality: Ultra High Pressure Homogenization (UHPH) and Pulsed Light (PL) (Table 2). Several recent reviews summarize the main features of UHPH (Zamora and Guamis, 2015; Patrignani and Lanciotti, 2016; Comuzzo and Calligaris, 2019; Morata and Guamis, 2020) and PL (Gómez-López et al., 2007; Oms-Oliu et al., 2010; Santamera et al., 2020; Vargas-Ramella et al., 2021).

UHPH involves a continuous pumping of liquid food (grape juice or wine) at pressure higher than 200 MPa (commonly 300 MPa) followed by a depressurization at atmospheric pressure through a special highly resistant valve (Zamora and Guamis, 2015; Morata and Guamis, 2020). In the valve, the fluid is subjected to extreme impact and shear stresses producing nanofragmentation of colloidal structures and microorganisms, down to a size of 300–500 nm (Morata and Guamis, 2020). This breakdown has a very powerful antimicrobial effect. It allows even the spores inactivation (depending on the in-valve temperature), but with a very gentle impact on sensory quality, thanks to the preservation of sensitive molecules such as terpenes (Bañuelos et al., 2020) and anthocyanins (Vaquero et al., 2022) and without formation of thermal markers such as hydroxymethylfurfural (Bañuelos et al., 2020). UHPH also inactivates oxidative enzymes (PPOs) and preserves the antioxidant activity (Loira et al., 2018a; Bañuelos et al., 2020). The highly effective elimination of wild microorganisms allows successful inoculations, even with non-*Saccharomyces* with low fermentative yield, and, therefore, permits a good expression of their metabolic profile, achieving good acidification and positive sensory impact.

PL entails the application of a high intensity broad spectrum light (200–2,500 nm) rich in UV (200–280 nm) by short duration light flashes (1 μs-0.1 s), typically using xenon lamps (Gómez-López et al., 2007; Santamera et al., 2020; Vargas-Ramella et al., 2021). The main drawback is the penetration depth which is less than 1 mm, but it can be applied on the surface of the grape after destemming to successfully eliminate wild yeasts (Escott et al., 2017). It therefore facilitates the implantation of unconventional yeast starters producing a high sensory impact on wine profile (Escott et al., 2021). In addition, PL is a gentle non-thermal technology (ΔT < 4°C) with protective effect on phenols and antioxidant capacity and low effects on color and anthocyanin degradation (Escott et al., 2017; Chakraborty et al., 2020; Bhagat and Chakraborty, 2022).

## Conclusion

The association of emerging biotechnologies such as the use of non-*Saccharomyces* yeasts in sequential or mixed fermentations and non-thermal technologies to control wild microorganisms and the activity of oxidative enzymes in grapes or must may be a powerful strategy to improve wine quality in warm areas. This can improve the quality of wines from neutral varieties or facilitate the expression of under-ripe grapes. The stability and time persistence of these wines is also improved by the microbial control through emerging non-thermal technologies, the bioprotection and acidification produced by some of the non-*Saccharomyces* species discussed above, allowing the reduction of SO_2_ levels. This can also be supported by using antimicrobial additives such as fumaric acid and by the antioxidant properties of yeast derivatives. A new enology for a new climate scenario.

## Author contributions

PC: Conceptualization, Writing – original draft, Writing – review & editing. JF: Writing – original draft, Writing – review & editing, Resources. SV: Writing – original draft, Writing – review & editing. IL: Writing – original draft, Writing – review & editing. AM: Writing – original draft, Writing – review & editing, Conceptualization, Funding acquisition, Validation.

## Footnotes

^1^ https://www.oiv.int/public/medias/1573/oiv-oeno-496-2013-en.pdf
